# Extracellular Hb Enhances Cardiac Toxicity in Endotoxemic Guinea Pigs: Protective Role of Haptoglobin

**DOI:** 10.3390/toxins6041244

**Published:** 2014-03-31

**Authors:** Jin Hyen Baek, Xiaoyuan Zhang, Matthew C. Williams, Dominik J. Schaer, Paul W. Buehler, Felice D’Agnillo

**Affiliations:** 1 Laboratory of Biochemistry and Vascular Biology, Division of Hematology, Center for Biologics Evaluation and Research, Food and Drug Administration, Bethesda, MD 20892, USA; E-Mails: jin.baek@fda.hhs.gov (J.H.B.); xiaoyuan.zhang@fda.hhs.gov (X.Z.); matthew.williams@fda.hhs.gov (M.C.W.); paul.buehler@fda.hhs.gov (P.W.B.); 2 Division of Internal Medicine, University Hospital, CH-8091 Zurich, Switzerland; E-Mail: dominik.schaer@usz.ch

**Keywords:** hemoglobin, endothelium, endotoxin, haptoglobin, myocardial injury

## Abstract

Endotoxemia plays a major causative role in the myocardial injury and dysfunction associated with sepsis. Extracellular hemoglobin (Hb) has been shown to enhance the pathophysiology of endotoxemia. In the present study, we examined the myocardial pathophysiology in guinea pigs infused with lipopolysaccharide (LPS), a Gram-negative bacterial endotoxin, and purified Hb. We also examined whether the administration of the Hb scavenger haptoglobin (Hp) could protect against the effects observed. Here, we show that Hb infusion following LPS administration, but not either insult alone, increased myocardial iron deposition, heme oxygenase-1 expression, phagocyte activation and infiltration, as well as oxidative DNA damage and apoptosis assessed by 8-hydroxy-2'-deoxyguanosine (8-OHdG) and terminal deoxynucleotidyl transferase dUTP nick end labeling (TUNEL) immunostaining, respectively. Co-administration of Hp significantly attenuated the myocardial events induced by the combination of LPS and Hb. These findings may have relevant therapeutic implications for the management of sepsis during concomitant disease or clinical interventions associated with the increased co-exposures to LPS and Hb, such as trauma, surgery or massive blood transfusions.

## 1. Introduction

Myocardial dysfunction and injury are key pathophysiological events in sepsis and septic shock [1,2]. Lipopolysaccharide (LPS), a Gram-negative bacterial endotoxin, is a primary mediator of the cardiovascular dysfunction associated with sepsis or septic shock [3,4]. The mechanisms underlying LPS-mediated cardiovascular dysfunction are multifactorial, but generally implicate the overproduction of cytokines (*i.e*., tumor necrosis factor-alpha, interleukin 1-beta) and reactive oxygen/nitrogen species as causative processes [3,4].

Several studies have demonstrated that LPS pathophysiology can be exacerbated by extracellular hemoglobin (Hb) [5,6,7]. In mice, intra-peritoneal injection of LPS combined with intravascular infusion of purified Hb induced greater mortality compared to Hb or LPS alone [5]. In rabbits, intravenous administration of chemically cross-linked Hb and LPS caused significantly greater cardiovascular dysfunction and mortality compared to Hb or LPS alone [6]. Murine models of infection coupled with a deficiency in heme oxygenase-1 (HO-1), a key enzyme of heme catabolism, suggest that the release of heme following hemolysis increases mortality [8]. In humans, the level of hemolysis occurring during Gram-negative bacteremia may be associated with an increased risk for mortality [9,10]. Plasma Hb levels in these patients were reported to occur over a range of concentrations from 10 to 40 mg/dL (equivalent to ~5 to 25 µM as heme) [9,10]. Reduced plasma levels of hemopexin and haptoglobin (Hp) also correlated with an increased risk for mortality in septicemia [11].

Hb enhancement of LPS pathophysiology may be particularly relevant in Gram-negative sepsis with concomitant trauma, surgery or blood transfusions when there is an increased likelihood for interaction between bacterial cell wall LPS and circulating extracellular Hb. Under such circumstances, it is postulated that the therapeutic management of Hb-LPS synergism may significantly ameliorate clinical outcomes. Hp is the Hb scavenger in the plasma of mammals, existing in humans as three primary phenotypes (Hp 1-1, Hp 2-1 and Hp 2-2) within a concentration range of 0.3–1.9 mg/mL [12,13]. These three Hp polymorphisms all contain the same Hb binding β globin (Hp^β^), but differ in their α globin (Hp^α1^ or Hp^α2^) composition [14,15]. This results in dimeric (Hp 1-1) or multimeric (Hp 2-1, Hp 2-2) forms, as denoted by the number of α globin cysteines involved in disulfide bond formation. Several studies have shown that Hp prevents Hb renal excretion and subsequent kidney injury following experimental and clinical hemolysis [16,17,18]. In the present study, we evaluated the acute myocardial responses of guinea pigs challenged with LPS and Hb with or without the co-administration of purified multimeric human Hp.

## 2. Results

### 2.1. Sequestration of Hb by Hp Prevents Renal Filtration

**Figure 1:**
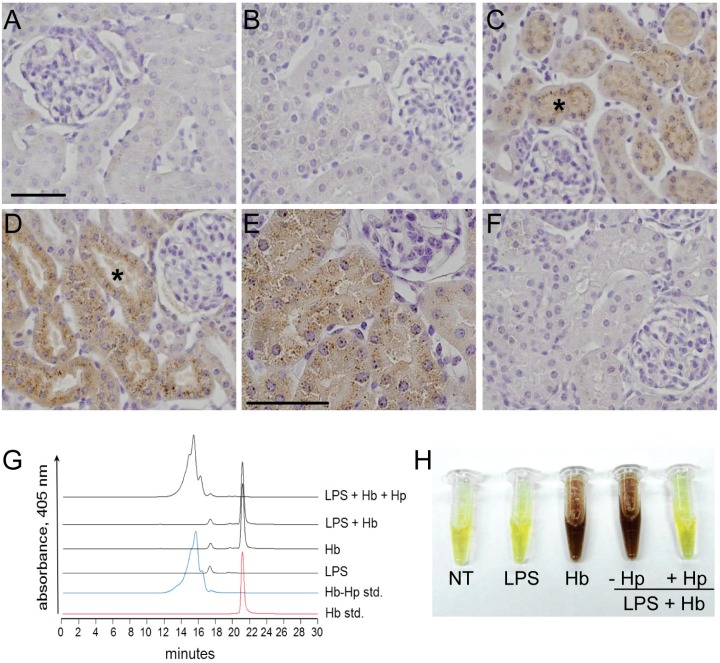
Hp prevents renal exposure to Hb. Renal non-heme iron deposition was not detectable in surgical control animals (**A**) or with LPS alone (**B**). Animals infused with Hb alone (**C**) or LPS + Hb (**D**,**E**) demonstrated increased iron deposits in the tubular epithelium of the renal cortex (brown staining). The asterisk in Panels C and D denote the lumen of a representative tubule containing iron deposits. Administration of Hp with Hb following LPS prevented iron deposition (**F**). Panels A–D and F, 400×; Panel E, 600×. (**G**) SEC-HPLC chromatographs of plasma samples, purified Hb standard (red trace), and a purified Hb-Hp standard (blue trace). (**H**) Representative images of urine samples 1 h after dosing. Scale bars = 50 µm.

A primary function of plasma Hp is to prevent renal excretion of Hb dimers [19]. To validate that the Hp administration conditions used in the present model effectively bound and maintained the extracellular Hb within a Hb-Hp complex, we first examined the renal deposition of non-heme iron as a marker of Hb exposure. Non-heme iron deposition was not observed in surgical control and LPS-infused animals using the Perls diaminobenzidine (DAB) intensification method (Figure 1A,B). However, Hb and LPS + Hb administered animals showed non-heme iron accumulation in the tubular epithelium of the renal cortex at 24 h post exposure. This was visualized as brown staining within hemosiderin granules (Figure 1C–E). Conversely, when LPS + Hb infusions were combined with concomitant Hp administration, no renal non-heme iron deposits were visualized (Figure 1F). 

These findings are supported by size exclusion (SEC)-HPLC analysis of plasma samples collected 1 h after dosing (Figure 1G). Representative samples from Hb and LPS + Hb administered animals demonstrated a single free Hb peak at an elution time of 21 min. This was confirmed by the elution time profile of the Hb standard solution. In the presence of Hp, Hb was completely bound and the elution time shifted to a broad band of peaks eluting over 13–17 min. This was also consistent with the elution time profile of the Hb-Hp standard. As a final confirmation, Hp sequestration of Hb was measured as the extent of hemoglobinuria over the initial hour after dosing (Figure 1H). Urinary Hb accumulation was only observed in Hb and LPS + Hb administered animals, but not in surgical controls, LPS or LPS + Hb + Hp administered animals. These data confirm that the Hp dose used in this study was effective in scavenging and maintaining all extracellular Hb within an Hp-bound complex. 

### 2.2. Hp Prevents Myocardial Iron Deposition and HO-1 Induction by LPS plus Hb

**Figure 2:**
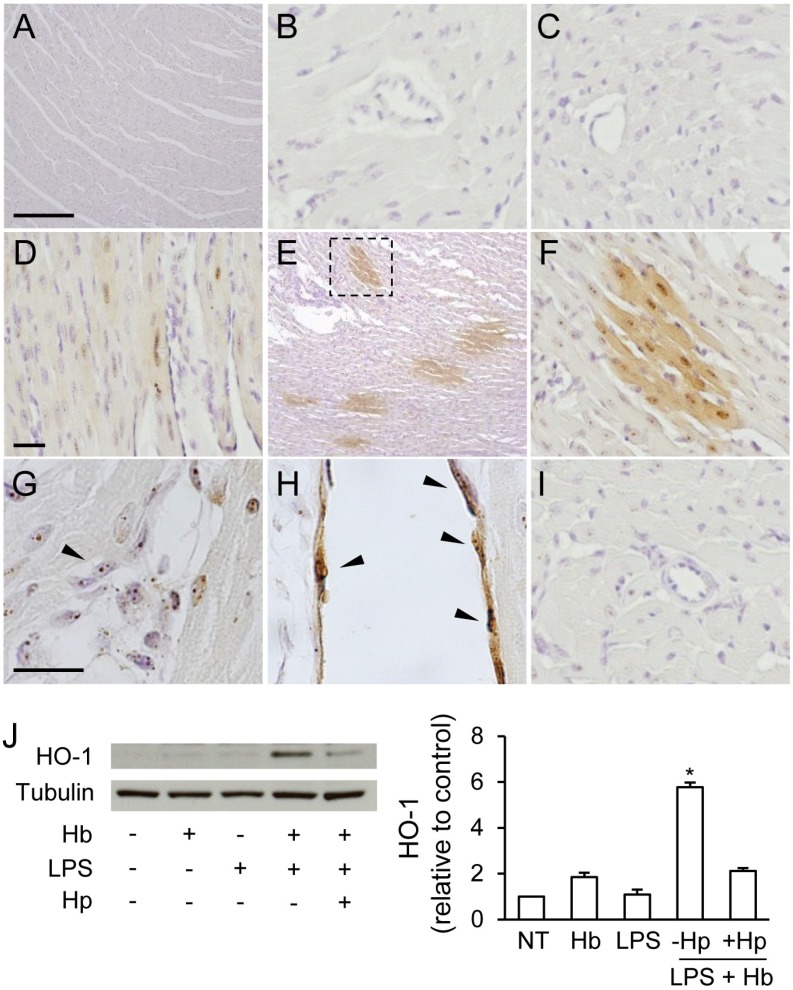
Myocardial non-heme iron staining in surgical control animals (**A**,**B**), LPS alone (**C**), Hb alone (**D**), LPS + Hb (**E-H**), and LPS + Hb + Hp (**I**). High magnification of the boxed region shows cytoplasmic and nuclear iron staining in myocytes (brown) and surrounding myocytes containing only punctate nuclear iron deposits (F). Arrowheads show iron deposits in the perivascular regions and endothelium of cardiac blood vessels (G,H) (600×). Scale bars = 200 µm (A and E) or 25 µm (B–D, F–I). (**J**) Myocardial HO-1 expression by Western blot. Representative blots for HO-1 and tubulin are shown. Densitometry values as a ratio to tubulin were normalized to non-treated controls (NT) and presented as the means ± SEM. * *p* < 0.05 *vs*. NT (*n* = 4 animals per group).

Non-heme iron deposition was not observed in cardiac sections from surgical control or endotoxemic animals (Figure 2A–C). Infusion with Hb alone produced minor, but detectable, iron deposition in focal myocytes (Figure 2D). Animals infused with LPS followed by Hb showed diffuse regions of iron accumulation within myocytes at 24 h post-Hb infusion (Figure 2E,F). Non-heme iron staining was also observed in endothelial cells and perivascular regions of small and medium-sized cardiac blood vessels (Figure 2G,H). Conversely, the administration of Hp with Hb in endotoxemic animals prevented iron deposition in the contractile tissue of the heart and within myocardial blood vessels (Figure 2I). These data suggest that Hp attenuates extravascular translocation of Hb and iron deposition within the myocardium of endotoxemic animals.

To further evaluate myocardial exposure to Hb, we examined HO-1 expression by Western blot. No significant changes in HO-1 expression were observed in animals infused with LPS or Hb alone (Figure 2J). In contrast, animals infused with LPS + Hb showed a six-fold increase in HO-1 expression 24 h post-infusion compared to surgical controls. Co-administration of Hp with Hb prevented the increase in HO-1 expression. 

**Figure 3:**
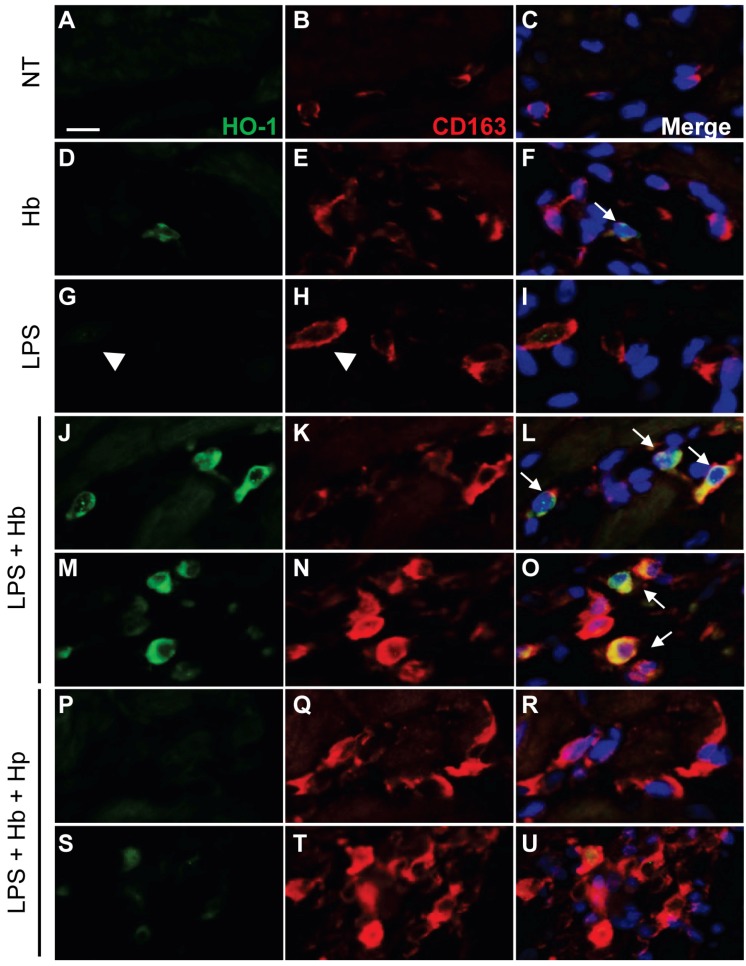
Immunofluorescence analysis of myocardial HO-1 and CD163 expression. Surgical non-treated (NT) control animals showed minimal HO-1 (green) expression in resident CD163^+^ perivascular macrophages (red) (**A**–**C**). Sporadic HO-1 expression (white arrow) localized to CD163^+^ cells observed with Hb alone (**D**–**F**). With LPS alone, CD163^+^ infiltrates and perivascular macrophages showed minimal HO-1 expression (white arrowhead) (**G**–**I**). Increased HO-1 expression localized to CD163^+^ infiltrates and perivascular macrophages observed with LPS + Hb (white arrows) (**J**–**L**,**M**–**O**). Hp reduces HO-1 expression in CD163^+^ infiltrates and perivascular cells (**P**–**R**,**S**–**U**). Nuclei were counterstained with Hoechst 33342 (blue). Scale bar = 10 µm.

To identify the HO-1-expressing cells, myocardial sections were double labeled for HO-1 and CD163, a marker of monocytes/macrophages. In surgical controls, CD163^+^ perivascular cells that showed no HO-1 reactivity were detectable as flattened and elongated cells around small to medium-sized blood vessels (Figure 3A–C). In Hb infused animals, CD163^+^ perivascular cells were primarily negative for HO-1, although a few cells with a low level HO-1 reactivity were detectable (Figure 3D–F). In endotoxemic animals, CD163^+^ perivascular cells showed no HO-1 reactivity (Figure 3G–I). In contrast, LPS + Hb animals showed an increased accumulation of CD163^+^/HO-1^+^ cells around and within small to medium-sized myocardial blood vessels (Figure 3J–L) and in the adventitial regions of large epicardial blood vessels (Figure 3M–O). Hp administration attenuated the induction of HO-1 among CD163^+^ cells in the myocardium (Figure 3P–R) and around large blood vessels (Figure 3S–U).

### 2.3. Hp Reduces Myocardial Myeloperoxidase Expression Induced by LPS plus Hb

**Figure 4:**
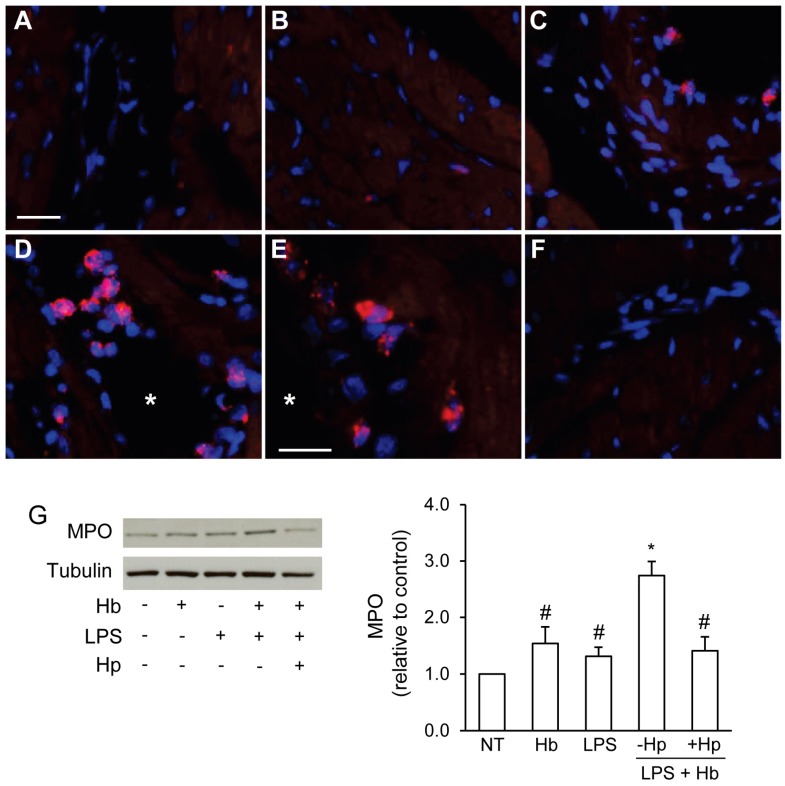
Immunofluorescence analysis of myocardial MPO expression in surgical control animals (**A**) and animals infused with Hb (**B**), LPS (**C**), LPS + Hb (**D**, **E**) or LPS + Hb + Hp (**F**). MPO^+^ infiltrates expressed red-stained azurophilic granules in the lumen (D) and subendothelial layers (E) of myocardial blood vessels. Asterisks in Panel D and E denote the lumen of a blood vessel. Nuclei were counterstained with Hoechst 33342 (blue). Scale bar = 20 µm (A–D, F) or 10 µm (E). (**G**) Myocardial MPO expression by Western blot. Representative blots for MPO and tubulin are shown. Densitometry values as a ratio to tubulin were normalized to NT and presented as the means ± SEM. * *p* < 0.05 *vs*. NT, # *p* < 0.05 *vs*. LPS + Hb (*n* = 4 animals per group).

We next examined the myocardial expression of myeloperoxidase (MPO), an enzyme found primarily in neutrophils and macrophages that is considered a reliable marker of inflammation and oxidative stress [20]. Immunofluorescence analysis of MPO showed minimal staining in surgical controls and Hb infused animals (Figure 4A,B). MPO^+^ cells were detected sporadically in large myocardial blood vessels of endotoxemic animals (Figure 4C). Animals infused with LPS + Hb showed a higher incidence of MPO^+^ infiltrates primarily in the lumen and subendothelial layers of blood vessels located in the ventricular myocardium (Figure 4D,E). Co-administration of Hp decreased the number of detectable MPO^+^ cells (Figure 4F). Western blot analysis of myocardial tissue extracts from animals infused with LPS or Hb alone showed no significant change in MPO expression compared to surgical controls (Figure 4G). In contrast, LPS + Hb induced a significant increase in MPO expression that was prevented with the co-administration of Hp.

### 2.4. Hp Protects against Myocardial Oxidative DNA Damage and Apoptosis Induced by LPS plus Hb

**Figure 5:**
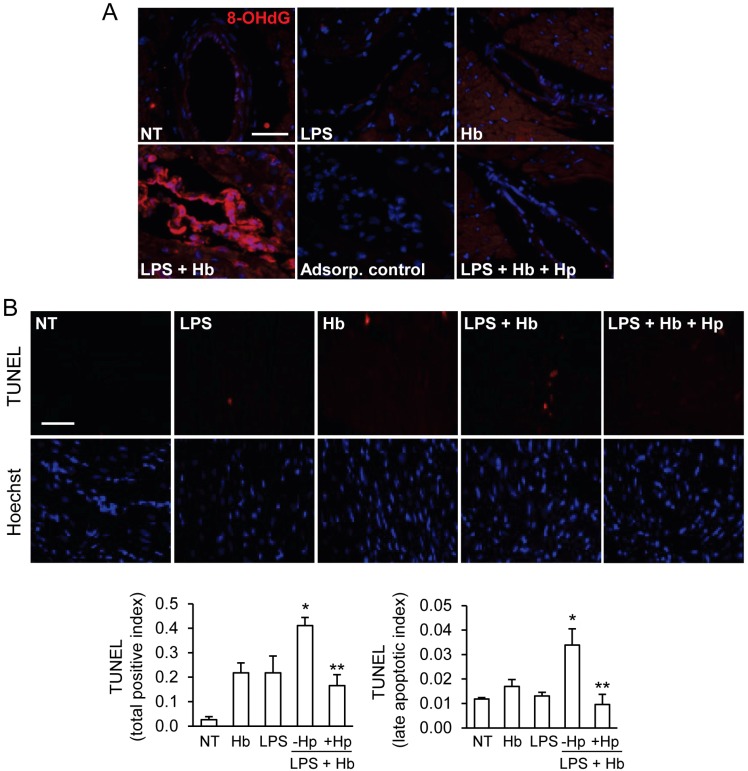
(**A**) Immunofluorescence analysis of 8-hydroxy-2'-deoxyguanosine (8-OHdG) in myocardial sections of surgical control animals (NT), LPS, Hb, LPS + Hb and LPS + Hb + Hp. Pre-incubation of primary antibody with 8-OHdG (adsorption control) completely blocked the positive staining associated with the same blood vessel depicted in the LPS + Hb panel. (**B**) Representative images of TUNEL staining (red) and Hoechst-stained nuclei are shown. Quantification of TUNEL staining (total positive cells and late apoptotic cells) was performed as described in the Experimental Section. Means ± SEM for each group are shown. * *p* < 0.05 *vs*. NT, ** *p* < 0.05 *vs*. LPS + Hb. Scale bar = 50 µm.

To further investigate oxidative stress and injury in this model, we analyzed 8-hydroxy-2'-deoxyguanosine (8-OHdG), a marker of oxidative DNA damage. Minimal 8-OHdG staining was detected in surgical controls or in animals infused with LPS or Hb alone (Figure 5A). Increased 8-OHdG reactivity was observed in animals infused with LPS + Hb, particularly in the endothelial lining of many medium- to small-sized vessels in the ventricular regions of the myocardium. In LPS + Hb, but not LPS alone, perivascular macrophages and infiltrates also showed increased 8-OHdG reactivity. An increase in 8-OHdG reactivity was also evident in myocytes located in the vicinity of 8-OHdG positive blood vessels. Hp co-administration markedly reduced the extent of 8-OHdG staining in cardiac blood vessels and within the myocardium (Figure 5A).

To further evaluate DNA damage and apoptosis, myocardial sections were analyzed by the TUNEL assay (Figure 5B). Two types of TUNEL positive nuclei were distinguishable and quantified separately in these sections. The total positive cell index represented the nuclei with detectable TUNEL reactivity that showed no discernible changes in nuclear morphology. The late apoptotic (or necrotic) index represented the TUNEL-positive nuclei with confirmed nuclear condensation, disintegration or apoptotic body formation. In general, TUNEL-positive nuclei were evident among both myocytes, and non-myocyte (*i.e*., perivascular macrophages, endothelial cells) populations though the proportion of TUNEL-positive cells with a normal nuclear appearance were significantly greater than the confirmed late apoptotic (necrotic) cells. Infusion with LPS or Hb alone did not increase the total positive or late apoptotic cells compared to surgical controls. In contrast, LPS + Hb increased TUNEL reactivity among both the total positive cells and late apoptotic cells (Figure 5B). Hp administration significantly reduced TUNEL positivity induced by LPS + Hb.

## 3. Discussion

Studies in animal models of endotoxemia have reported that extracellular Hb exposure significantly increases lethality and may be associated with cardiovascular dysfunction [5,6]. The synergism between Hb and LPS is often attributed to Hb binding to LPS, causing disaggregation of LPS multimers and enhancing the exposure to LPS lipid A moieties [21,22]. Previous studies have also suggested that Hb augments LPS-induced TNF-alpha response in cultured monocytes and *in vivo*, an effect thought to contribute to the synergistic toxicity of Hb and LPS [23,24]. Enzymes, such as xanthine oxidase, phagocyte NADPH oxidase and MPO, have also been implicated in the overproduction of reactive oxygen species in sepsis and endotoxemia [25,26,27,28,29]. Several studies have investigated the role of MPO in monitoring cardiovascular disease primarily related to the inflammation observed in atherosclerosis [20], while the DNA oxidative product, 8-OHdG, is reported to be a relevant marker of sepsis-related oxidative stress and outcome [30]. In the context of the present study, we postulate that the oxidative microenvironment created by activated circulating inflammatory cells or tissue resident cells along the endothelial lining and within perivascular sites could be a major factor driving the oxidative reactions of Hb or its breakdown products [31]. Consistent with this view, the enhanced redox activity of various Hbs was shown to dramatically enhance LPS-induced apoptosis in endothelial cell culture [32]. Endothelial dysfunction is a hallmark of sepsis, which is often characterized by increased vascular permeability induced by LPS-mediated processes in several tissues, including the right and left ventricular coronary micro-vasculature [33,34]. Thus, LPS-induced loss of barrier function may represent a contributing mechanism underlying the extravasation of extracellular Hb or its breakdown products into perivascular sites.

Consistent with these concepts, the present findings suggest that extracellular Hb exposure in endotoxemic guinea pigs can exacerbate inflammation, oxidative stress and cellular injury. These events are likely mediated by increased vascular permeability and perivascular myocardial exposure to free Hb or its breakdown products. We show that Hb infusion following LPS administration, but not either insult alone, increased myocardial iron deposition and HO-1 expression supporting the extravascular exposure to Hb with subsequent heme release. Enhanced MPO- and 8-OHdG-positive infiltrates, as well as 8-OHdG-positive perivascular and endothelial cells of myocardial blood vessels are supportive of increased inflammation and oxidative stress. This is also consistent with the increased TUNEL reactivity observed in myocyte and non-myocyte populations.

Hp is an important acute phase protein that initially increases in response to inflammation and infection. Other Hb detoxification proteins within this class also include hemopexin and ferritin, which participate in heme clearance and intracellular iron storage, respectively. Additional acute phase reactants relevant to infection and endotoxemia include: C-reactive protein, clotting factors, hepcidin and complement that participates in bacterial opsonization [35]. Our results demonstrate that the co-administration of Hp blocked the myocardial events induced by the combination of LPS and Hb. Several mechanisms may explain the protective activity of Hp. First, Hp may limit the translocation of Hb across the hyperpermeable endothelium. Second, Hp may prevent the release of heme into the myocardium and the vasculature, and third, Hp may control the redox activity of Hb driven by inflammation-derived hydro- and lipo-peroxides [36,37]. Together, the present data support a potentially useful therapeutic role for Hp in clinical or disease settings associated with the increased co-exposure to Hb and LPS, such as sepsis with concomitant trauma, surgery or blood transfusions.

## 4. Experimental Section

### 4.1. Reagents and Antibodies

Stroma-free human Hb was purified from hemolysate to remove catalase by ion exchange chromatography on a diethylaminoethanol (DEAE) Sephadex column (GE-Healthcare, Sweden) and concentrated to 150 mg/mL in normal saline. Human multimeric phenotype specified product, absent dimeric Hp, was provided by Benesis Corporation (Osaka, Japan) at a concentration of 20 mg/mL in normal saline. Hp was then concentrated to 60 mg/mL. Standards of Hb-Hp complex for analytical size exclusion chromatography were separated from excessive (non-bound) Hb by gel chromatography on a HiLoad 26/60 Superdex 200 chromatography column (GE Healthcare Life Sciences, Piscataway, NJ, USA) and buffer exchanged into 50 mM phosphate-buffered saline (PBS). Rabbit polyclonal antibody to HO-1 was purchased from Enzo Life Sciences (Farmingdale, NY, USA). Mouse anti-CD163 clone EDHu-1 was obtained from AbD Serotec (Raleigh, NC, USA). Rabbit polyclonal anti-tubulin was obtained from Santa Cruz Biotechnologies (Santa Cruz, CA, USA). Rabbit polyclonal antibody to MPO was purchased from Cell Signaling Technology (Danvers, MA, USA). Rabbit monoclonal antibody to MPO was obtained from Abcam (Cambridge, MA, USA). Mouse monoclonal antibody to 8-OHdG (clone N45.1) was purchased from Oxis International (Foster City, CA, USA).

### 4.2. Animal Experimental Protocol

Male Hartley guinea pigs (Charles Rivers Laboratories, Wilmington, MA, USA) were acclimated for one week upon arrival to the Food and Drug Administration (FDA) Center for Biologics Evaluation and Research (CBER) animal care facility. All animals weighed 400–450 g before surgery. Animal protocols were approved by the FDA CBER Institutional Animal Care and Use Committee, with all experimental procedures performed in adherence to the National Institutes of Health guidelines on the use of experimental animals. Surgical preparation was performed as previously described for implantation of indwelling venous and arterial catheters. After a 24 h period of post-surgical recovery, animals were randomized to one of five groups as follows: (1) surgical non-treated control; (2) LPS (10 mg/kg, i.v.); (3) Hb (150 mg, i.v.); (4) LPS + Hb; and (5) LPS + Hb + Hp (150 mg Hb + 180 mg Hp, to ensure complete binding of Hb). All groups received equal infusion volumes normalized with 0.9% NaCl. LPS was injected via an indwelling jugular catheter 2 h prior to administration of saline control, Hb or Hb + Hp. This LPS challenge produced sublethal endotoxemia allowing for the evaluation of Hb and Hb + Hp effects in the absence of mortality. In the three Hb infusion groups, the maximal plasma levels of Hb measured as heme equivalents were approximately 150 µM (260 mg/dL Hb).

Blood (0.2 mL) was collected at baseline and after administration of control or test solution as follows: T = 0 (end administration), 0.5, 1, 2, 4, 6, 12 and 24 h (study termination). Urine was collected over the initial 4 h following administration of test or control solutions and at 24 h (study termination). Tissue harvesting was performed at 24 h following intraperitoneal administration of Euthasol (0.22 mg/kg) to induce deep anesthesia. Whole body perfusion was performed with 60 mL of cold 0.9% NaCl to remove blood from major organ systems. Heart was cut into two longitudinal sections starting at the apex through the septum to the aortic root to expose the right and left ventricular walls and the septum. Similarly, right and left kidneys were cut into two longitudinal sections to expose the cortex and medulla. Half of the tissue was fixed in formalin, and half was snap frozen in liquid nitrogen.

### 4.3. Plasma Hemoglobin Distribution

Following administration, Hp bound and unbound fractions of Hb were determined by size exclusion high performance liquid chromatography (SEC-HPLC) using a Waters 600 controller, a Waters 600 pump and a Waters 2499 photodiode array detector (Waters, Corp, Millford, MA, USA). Plasma samples were separated on a BioSep-SEC-S3000 (600 × 7.5 mm) column (Phenomenex, Torrance, CA, USA) with 50 mM potassium phosphate, pH 7.4 as the mobile phase. Column injections were maintained at consistent volumes using a 50-μL injection loop. All samples were monitored at λ = 280 nm and λ = 405 nm in dual wavelength mode. Hb bound and unbound in plasma was determined by dividing the Hb peak area and the Hb-Hp peak area by additive areas under the Hb-Hp chromatographic peak (13–17 min elution) and the Hb chromatographic peak (21 min elution) at λ = 405 nm.

### 4.4. Non-heme Iron Immunohistochemistry

Hearts and kidneys were fixed in 10% formalin for 24 h, then stored in 100% isopropanol, embedded in paraffin, and 5 µm sections were prepared. Non-heme ferric iron deposition was detected using Perls’ method with DAB intensification. Sections were dewaxed in Safe Clear™ (Houston, TX, USA) and rehydrated in graded ethanol and deionized water. Tissues were then incubated with Perls’ iron reagent containing 5% potassium ferrocyanide and 2% hydrochloric acid for 45 min at room temperature and rinsed in deionized water. Sections were then incubated with 0.3% hydrogen peroxide and 0.01 M sodium azide in methanol for 30 min at room temperature. All sections were then rinsed in 0.1 M phosphate buffer, pH 7.4, incubated with DAB for three minutes, washed in deionized water and lightly counterstained with Gill’s II hematoxylin. After dehydrating in graded ethanol and Safeclear, slides were mounted in Permount and coverslipped. Images were obtained using an Olympus IX71 inverted microscope.

### 4.5. Preparation of Heart Tissue Lysates

Frozen heart samples from the ventricular compartment were dounce-homogenized in the presence of ice-cold modified radioimmunoprecipitation assay (RIPA) buffer (50 mM Tris, 150 mM sodium chloride, 1% IgePal^®^-630, 0.5% sodium deoxycholate, 1 mM ethylenediaminetetraacetic acid (EDTA) containing protease inhibitors (Cocktail Set III, Calbiochem, CA, USA). Lysates were centrifuged at 10,000 rpm at 4 °C for 30 min. Supernatants were collected and stored at −80 °C. Protein concentrations were measured using the bicinchoninic acid (BCA) protein assay (Thermo Scientific, Rockford, IL, USA).

### 4.6. Western Blot Analyses

Heart lysates were resolved on 4%–12% Bis-Tris gels, transferred to polyvinylidene fluoride (PVDF) membranes and blocked for 1 h in Tris buffered saline with 0.1% Tween-20 (TBS-T) and 5% nonfat dry milk. Membranes were incubated overnight at 4 °C with antibodies to HO-1 (1:2500) and MPO (1:2000) in TBS-T with 1% nonfat dry milk or 3% bovine serum albumin (for MPO), washed and then incubated with a relevant horseradish peroxidase (HRP)-conjugated secondary antibody for 1 h. The signal was developed using the ECL Plus chemiluminescence kit (Amersham, Arlington Heights, IL) and detected on HyperECL film. Densitometry analysis was performed using ImageJ software (National Institutes of Health, Bethesda, MD, USA) with normalization to tubulin.

### 4.7. Immunofluorescence

Paraffin-embedded slides were dewaxed and rehydrated. Slides were heat-treated in a microwave oven for 15 min in 10 mM sodium citrate buffer, pH 6.0, cooled for 30 min at room temperature and rinsed with deionized water and PBS-T. Sections were then blocked in PBS-T with 5% goat or horse serum for 1 h at room temperature followed by overnight incubation at 4 °C with antibodies to HO-1, MPO, CD163 and 8-OHdG. Sections were rinsed and incubated with relevant Alexa-Fluor 488- and Alexa-Fluor 555-conjugated secondary antibodies for 1 h at room temperature. Nuclei were counterstained with Hoechst 33342. For double labeling experiments, primary antibodies were mixed together and incubated overnight at 4 °C. Sections stained with secondary antibodies alone showed no specific staining. Images were processed using an Olympus IX71 inverted microscope equipped with an Olympus DP70 digital camera.

### 4.8. TUNEL Assay

Hearts were removed and fixed in neutral buffered 10% formalin at room temperature for 24 h before embedding in paraffin and sectioning. Sections were deparaffinized, antigen retrieved with proteinase K treatment (20 µg/mL for 20 min at room temperature) and then subjected to TUNEL (terminal deoxynucleotidyl transferase-mediated dUTP nick end labeling) staining according to the manufacturer’s instructions (Apotag Red In Situ Apoptosis Detection kit, Millipore, Billerica, MA, USA). Negative controls performed in the absence of the terminal deoxynucleotidyl transferase TdT enzyme showed no TUNEL reactivity. The TUNEL-positive nuclei and the total number of nuclei were counted in 6 fields of view per slide captured at 400× magnification. The average number of nuclei counted per field for each group was as follows; non-treated controls (175 ± 4), Hb (170 ± 13), LPS (162 ± 17), LPS + Hb (179 ± 7) and LPS + Hb +Hp (188 ± 10). The TUNEL-positive cell index was calculated as the number of nuclei with detectable TUNEL reactivity regardless of staining intensity or nuclear morphology divided by the total number of nuclei. The late apoptotic index was calculated as the number of TUNEL-positive nuclei with confirmed nuclear condensation or disintegration divided by the total number of nuclei.

### 4.9. Statistical Analysis

Data are represented as the means ± SEM for replicate experiments. Statistical analysis was performed by one-way ANOVA with a post-hoc Bonferroni’s test for all normally distributed, equal variance and equal number comparisons. A non-parametric one-way ANOVA was performed for comparisons with unequal distributions and non-matched group sizes. All analyses were performed using GraphPad Prism version 5 software. A *p*-value < 0.05 was considered statistically significant.

## 5. Conclusions

Extracellular Hb enhances the markers of iron deposition, inflammation, oxidative stress, and cell death in the myocardium of endotoxemic guinea pigs. The co-administration of Hp, a Hb scavenger protein, attenuated the myocardial events observed. These findings suggest that the synergistic toxicity induced by the co-exposure to LPS and Hb may be counteracted by therapeutic approaches designed to prevent or eliminate the accumulation of free Hb. This may have important implications for the management of sepsis and/or septic shock with concomitant trauma, surgery, or blood transfusions.

## References

[1] Zanotti-Cavazzoni S.L., Hollenberg S.M. (2009). Cardiac dysfunction in severe sepsis and septic shock. Curr. Opin. Crit Care.

[2] Flynn A., Chokkalingam Mani B., Mather P.J. (2010). Sepsis-induced cardiomyopathy: A review of pathophysiologic mechanisms. Heart Fail Rev..

[3] Balija T.M., Lowry S.F. (2011). Lipopolysaccharide and sepsis-associated myocardial dysfunction. Curr. Opin. Infect. Dis..

[4] Suliman H.B., Welty-Wolf K.E., Carraway M., Tatro L., Piantadosi C.A. (2004). Lipopolysaccharide induces oxidative cardiac mitochondrial damage and biogenesis. Cardiovasc. Res..

[5] Su D., Roth R.I., Yoshida M., Levin J. (1997). Hemoglobin increases mortality from bacterial endotoxin. Infect. Immun..

[6] Krishnamurti C., Carter A.J., Maglasang P., Hess J.R., Cutting M.A., Alving B.M. (1997). Cardiovascular toxicity of human cross-linked hemoglobin in a rabbit endotoxemia model. Crit. Care Med..

[7] McGahan M.C., Grimes A.M., Fleisher L.N. (1996). Hemoglobin exacerbates the ocular inflammatory response to endotoxin. Graefes Arch. Clin. Exp. Ophthalmol..

[8] Larsen R., Gozzelino R., Jeney V., Tokaji L., Bozza F.A., Japiassu A.M., Bonaparte D., Cavalcante M.M., Chora A., Ferreira A. (2010). A central role for free heme in the pathogenesis of severe sepsis. Sci. Transl. Med..

[9] Adamzik M., Hamburger T., Petrat F., Peters J., de Groot H., Hartmann M. (2012). Free hemoglobin concentration in severe sepsis: Methods of measurement and prediction of outcome. Crit. Care.

[10] Janz D.R., Bastarache J.A., Peterson J.F., Sills G., Wickersham N., May A.K., Roberts L.J., Ware L.B. (2013). Association between cell-free hemoglobin, acetaminophen, and mortality in patients with sepsis: An observational study. Crit. Care Med..

[11] Janz D.R., Bastarache J.A., Sills G., Wickersham N., May A.K., Bernard G.R., Ware L.B. (2013). Association between haptoglobin, hemopexin and mortality in adults with sepsis. Crit. Care.

[12] Smithies O., Connell G.E., Dixon G.H. (1962). Inheritance of haptoglobin subtypes. Am. J. Hum. Genet..

[13] Nosslin B.F., Nyman M. (1958). Haptoglobin determination in diagnosis of haemolytic diseases. Lancet.

[14] Andersen C.B., Torvund-Jensen M., Nielsen M.J., de Oliveira C.L., Hersleth H.P., Andersen N.H., Pedersen J.S., Andersen G.R., Moestrup S.K. (2012). Structure of the haptoglobin-haemoglobin complex. Nature.

[15] Connell G.E., Dixon G.H., Smithies O. (1962). Subdivision of the three common haptoglobin types based on “hidden” differences. Nature.

[16] Boretti F.S., Buehler P.W., D’Agnillo F., Kluge K., Glaus T., Butt O.I., Jia Y., Goede J., Pereira C.P., Maggiorini M. (2009). Sequestration of extracellular hemoglobin within a haptoglobin complex decreases its hypertensive and oxidative effects in dogs and guinea pigs. J. Clin. Invest.

[17] Lim S.K., Kim H., Lim S.K., bin Ali A., Lim Y.K., Wang Y., Chong S.M., Costantini F., Baumman H. (1998). Increased susceptibility in Hp knockout mice during acute hemolysis. Blood.

[18] Hashimoto K., Nomura K., Nakano M., Sasaki T., Kurosawa H. (1993). Pharmacological intervention for renal protection during cardiopulmonary bypass. Heart Vessel..

[19] Bunn H.F., Jandl J.H. (1969). The renal handling of hemoglobin. II. Catabolism. J. Exp. Med..

[20] Schindhelm R.K., van der Zwan L.P., Teerlink T., Scheffer P.G. (2009). Myeloperoxidase: A useful biomarker for cardiovascular disease risk stratification?. Clin. Chem..

[21] Kaca W., Roth R.I., Levin J. (1994). Hemoglobin, a newly recognized lipopolysaccharide (LPS)-binding protein that enhances LPS biological activity. J. Biol. Chem..

[22] Bahl N., Du R., Winarsih I., Ho B., Tucker-Kellogg L., Tidor B., Ding D.L. (2011). Delineation of lipopolysaccharide (LPS)-binding sites on hemoglobin: From in silico predictions to biophysical characterization. J. Biol. Chem..

[23] Bodet C., Chandad F., Grenier D. (2007). Hemoglobin and LPS act in synergy to amplify the inflammatory response. J. Dent. Res..

[24] Gorczynski R.M., Alexander C., Bessler W., Fournier K., Hoffmann P., Mach J.P., Manuel J., Ramakrishna V., Rietschel E.T., Song L. (2004). Characterization of an interaction between fetal hemoglobin and lipid A of LPS resulting in augmented induction of cytokine production in vivo and in vitro. Int. Immunopharmacol..

[25] Luchtemberg M.N., Petronilho F., Constantino L., Gelain D.P., Andrades M., Ritter C., Moreira J.C., Streck E.L., Dal-Pizzol F. (2008). Xanthine oxidase activity in patients with sepsis. Clin. Biochem..

[26] Wu J., Xu H., Yang M., Martin C.M., Kvietys P.R., Rui T. (2009). NADPH oxidase contributes to conversion of cardiac myocytes to a proinflammatory phenotype in sepsis. Free Radic. Biol. Med..

[27] Kothari N., Keshari R.S., Bogra J., Kohli M., Abbas H., Malik A., Dikshit M., Barthwal M.K. (2011). Increased myeloperoxidase enzyme activity in plasma is an indicator of inflammation and onset of sepsis. J. Crit. Care.

[28] Gao M., Ha T., Zhang X., Liu L., Wang X., Kelley J., Singh K., Kao R., Gao X., Williams D. (2012). Toll-like receptor 3 plays a central role in cardiac dysfunction during polymicrobial sepsis. Crit. Care Med..

[29] Huet O., Dupic L., Harrois A., Duranteau J. (2011). Oxidative stress and endothelial dysfunction during sepsis. Front. Biosci..

[30] Cheng W.E., Shih C.M., Hang L.W., Wu K.Y., Yang H.L., Hsu W.H., Hsia T.C. (2007). Urinary biomarker of oxidative stress correlating with outcome in critically septic patients. Intensive Care Med..

[31] Buehler P.W., D’Agnillo F. (2010). Toxicological consequences of extracellular hemoglobin: biochemical and physiological perspectives. Antioxid. Redox. Signal..

[32] D’Agnillo F. (2004). Redox active hemoglobin enhances lipopolysaccharide-induced injury to cultured bovine endothelial cells. Am. J. Physiol. Heart Circ. Physiol..

[33] Langheinrich A.C., Ritman E.L. (2006). Quantitative imaging of microvascular permeability in a rat model of lipopolysaccharide-induced sepsis: Evaluation using cryostatic micro-computed tomography. Invest Radiol..

[34] Darwish I., Liles W.C. (2013). Emerging therapeutic strategies to prevent infection-related microvascular endothelial activation and dysfunction. Virulence.

[35] Sankar V., Webster N.R. (2013). Clinical application of sepsis biomarkers. J. Anesth..

[36] Banerjee S., Jia Y., Siburt C.J., Abraham B., Wood F., Bonaventura C., Henkens R., Crumbliss A.L., Alayash A.I. (2013). Haptoglobin alters oxygenation and oxidation of hemoglobin and decreases propagation of peroxide-induced oxidative reactions. Free Radic. Biol. Med..

[37] Buehler P.W., Abraham B., Vallelian F., Linnemayr C., Pereira C.P., Cipollo J.F., Jia Y., Mikolajczyk M., Boretti F.S., Schoedon G. (2009). Haptoglobin preserves the CD163 hemoglobin scavenger pathway by shielding hemoglobin from peroxidative modification. Blood.

